# EFFECTS OF REMINISCENT MUSIC THERAPY WITH ROBOT-ASSISTED REHABILITATION FOR POSTSTROKE RECOVERY OLDER CHINESE

**DOI:** 10.1093/geroni/igae098.0350

**Published:** 2024-12-31

**Authors:** Qian Liu, Li Liu, Huiping Wu, Yujin Liu

**Affiliations:** Innovation Center of Nursing Research and Nursing Key Laboratory of Sichuan Province, West China Hospital, Sichuan University, Chengdu, Sichuan, China (People’s Republic); Innovation Center of Nursing Research and Nursing Key Laboratory of Sichuan Province, West China Hospital, Sichuan University, Chengdu, Sichuan, China (People’s Republic); Shenzhen Nurses Association, Shenzhen, Guangdong, China (People’s Republic); Changchun Humanities and Sciences College, Changchun, Jilin, China (People’s Republic)

## Abstract

This study aimed to investigate the impacts of reminiscent music therapy combined with robot-assisted rehabilitation on older stroke patients’ rehabilitation self-efficacy, activities of daily living, self-esteem, positive emotion, and upper limb function. A single-blind, three-arm randomized controlled trial was conducted in a Chinese tertiary hospital from November 2022 to January 2024. Experimental group A received reminiscent music therapy combined with robot-assisted rehabilitation and usual care, experimental group B received robot-assisted rehabilitation and usual care, and the control group only received usual care. Songs released from 1935 to 1980 were obtained via three music apps (QQ Music, KuGou and NetEase) commonly used in China. Robot-assisted rehabilitation with five game modes was carried out by occupational therapists. Outcomes included rehabilitation self-efficacy, activities of daily living, self-esteem, positive emotion, and upper limb function, which were evaluated at baseline and post intervention. A total of 126 older stroke patients completed the study. Baseline balance was established across the three groups. After the intervention, participants in the experimental group A reported greater rehabilitation self-efficacy, activities of daily living, self-esteem, positive emotion, and upper limb function than did those in experimental group B and the control group (p < 0.05). All outcome measurements were significantly greater than those at baseline (p < 0.05). Reminiscent music therapy combined with robot-assisted rehabilitation is a promising method for improving mental health and rehabilitation outcomes in older stroke patients. It may be incorporated in routine rehabilitation training.

